# First Report of Brucella Seroprevalence in Wild Boar Population in Serbia

**DOI:** 10.3390/vetsci9100575

**Published:** 2022-10-17

**Authors:** Zorana Zurovac Sapundzic, Jadranka Zutic, Natasa Stevic, Vesna Milicevic, Marina Radojicic, Slavoljub Stanojevic, Sonja Radojicic

**Affiliations:** 1 Scientific Institute of Veterinary Medicine of Serbia, Janisa Janulisa 14, 11000 Belgrade, Serbia; 2 Department of Infectious Animal Diseases and Diseases of Bees, Faculty of Veterinary Medicine, University of Belgrade, Bulevar Oslobodenja 18, 11000 Belgrade, Serbia; 3 Department of Microbiology and Immunology, Faculty of Veterinary Medicine, University of Belgrade, Bulevar Oslobodenja 18, 11000 Belgrade, Serbia; 4 Directorate of National Reference Laboratories, Batajnicki Drum 10, 11080 Zemun, Serbia

**Keywords:** wild boar, *Brucella*, Rose Bengal Test, ELISA, seroprevalence, Serbia

## Abstract

**Simple Summary:**

The presented manuscript provides the first data about the important zoonotic disease, brucellosis, in the population of wild boars on the territory of Serbia. Brucellosis is an important disease of animals, both domestic and wild, and humans, and is of exceptional importance. Recently, the disease has re-emerged in some countries and is a threat to public health. The infection was never investigated before in the population of wild boars in Serbia, although the reported infections in domestic pigs indicate the possible pathogen transmission from wild to domestic pigs. Applied serology assays provided *Brucella* seroprevalences in wild boars, while a wealth of statistics delivered important data. The obtained results confirm the presence of the infection in the population of wild boars in Serbia and open new chapters for the future investigations of brucellosis in wild boars in Serbia.

**Abstract:**

Brucellosis is one of the most important bacterial zoonotic diseases worldwide, characterized in domestic animals by long-term reproductive disorders. As known, wild boars (*Sus scrofa*) are natural hosts for *Brucella suis* biovar 2, in which the infection passes in inapparent form, increasing the pathogen transmission risk to domestic pigs, other domestic animals and humans. So far, no studies regarding brucellosis in wild boars in Serbia have been published. During the hunting season 2020/2021, 480 sera of wild boars living in Serbia were collected and tested for the presence of anti-*Brucella* antibodies. For the serological survey, the Rose Bengal Test (RBT) and competitive enzyme-linked immunosorbent assay (c-ELISA) were used. Of the 480 sera, 45 sera tested positive, indicating the acquired *Brucella* seroprevalence in wild boars of 9.4%. The greatest numbers of *Brucella* seropositive animals were detected in the eastern parts of the country and in one of the central districts, i.e., Pomoravski, Branicevski, Borski and Juznobanatski. This study provides the first data regarding brucellosis in the wild boar population in Serbia, revealing the seroprevalence of *Brucella*, thus indicating that wild boars as natural hosts and/or vectors of *Brucella* likely present a risk for the infection of other animals.

## 1. Introduction

Brucellosis is one of the most important contagious diseases of animals, including humans, as the disease has been registered on almost every continent in the past and is still one of the most significant bacterial zoonoses. *Brucella* is a group of small facultative, intracellular, Gram-negative, non-capsulated and non-motile coccobacilli. Within the genus *Brucella*, twelve species have been recognised so far. Beside the six well known species, i.e., the so-called classical *Brucella* species, *B. abortus*, *B. melitensis*, *B. ovis*, *B. suis*, *B. canis* and *B. neotomae*, novel classification involves *B. ceti*, *B. pinnipedialis*, *B. microti*, *B. inopinata* [1], *B. vulpis* [2], and last reported *B. papionis* [3]. The number of species illustrates the large variety of animals, also including wildlife, predisposed to infection with *Brucella*.

The disease in pigs is dominantly caused by *B. suis*, within which five different biovars are identified. *B. suis* biovars 1 and 3 circulate in the population of domestic pigs, biovar 2 in wild boars, biovar 4 in reindeer and biovar 5 in rodents.

Wild boars (*Sus scrofa*) are the natural hosts and reservoirs for numerous infectious diseases and zoonoses, including brucellosis [4]. The population of wild boars in Europe is constantly increasing, primarily due to the absence of natural predators combined with artificial feeding, high reproductive potential etc., [5] with estimated population density throughout Europe of up to 15 individuals/km^2^ [6]. As *B. suis* is considered practically eradicated on commercial pig farms across Europe, USA, Canada and Australia, wild boars represent a constant risk for the reintroduction of brucellosis into domestic pig population. Additionally, wild boars also represent a risk for the infection of cattle, being consequently hazardous for public health. Limited ways for decreasing the prevalence of brucellosis in natural reservoirs, as well as the high zoonotic potential of certain biovars, indicate that *B. suis* still owns an important place on the list of infectious agents. Although wild boars together with European hares (*Lepus europaeus*) are natural hosts for *B. suis* biovar 2, infections with other *Brucella* species (*B. melitensis* and *B. abortus*) were also registered [7]. *B. microti* was notably isolated from the submandibular lymph node of wild boar, although it is known that reservoirs for *B. microti* are voles [8], while the bacteria were also found in foxes [9] and soil [10].

The reported prevalence of brucellosis in wild boars is estimated as ranging from 8 to 32% throughout Europe [11]. On the other hand, using serological investigations, researchers reported *Brucella* seroprevalence in wild boars ranged from 0 to almost 60% [12,13,14,15,16].

Brucellosis in pigs is characterized by long-term reproductive disorders. However, the infection in wild boars is considered to be inapparent, without characteristic clinical symptoms or pathomorphological lesions, despite the successful isolation of the pathogen. The basis for this can lie in the low pathogenicity of *B. suis* biovar 2 for wild boars and/or in other as yet undefined reasons. The spread of infection is mostly via mating or by ingestion of contaminated feed or water.

Brucellosis can be diagnosed in two ways: directly via isolation of *Brucella*, or indirectly by detection of specific antibodies. Due to the required laboratory working conditions (biosafety level 3), the risk to laboratory staff’s health and frequent failures to isolate bacteria, classical microbiological methods are rarely performed. Serological investigations in wildlife are the most frequently carried out for screening purposes, using the Rose Bengal Test (RBT) together with the complement fixation test (CFT), both recommended as general-purpose diagnostic tests in all wildlife species. Nevertheless, enzyme-linked immunosorbent *assay* (*ELISA*), as a more reliable test, appeared to be useful for epidemiological serosurveys [7].

In Serbia, *B. melitensis*, *B. canis* and *B. suis* biovar 2 have been isolated so far [17]. To the best of our knowledge, there are no published data regarding brucellosis in wild boars in Serbia. Hence, the lack of data regarding brucellosis in wild boars hinders understanding of the potential spread of this infection to domestic pigs, other domestic animals and humans. Therefore, our study aimed to investigate the presence of anti-*Brucella* antibodies in wild boar sera and, thus, provide the first information about this significant infection in the population of wild boars in Serbia.

## 2. Materials and Methods

### 2.1. Sample Collection

**Figure 1:**
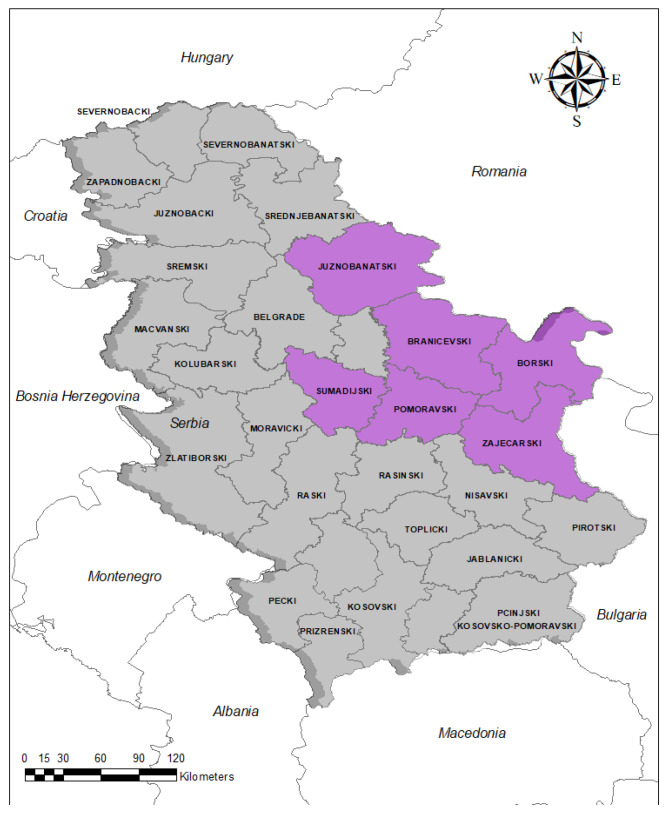
Administrative districts in Serbia where the tested wild boar sera were collected (purple areas).

The samples were collected during 2020 and 2021 from hunted wild boars living in six different administrative districts in Serbia: Branicevski, Borski, Zajecarski, Pomoravski, Juznobanatski and Sumadijski (Figure 1).

All of the wild boars were shot by registered hunters during the annual hunting season for wild boars. Age determination was conducted according to SCHEDA Ecological Associates, Inc. based on the number of permanent molars (one molar 6–18 months, two molars 1.5–2.5 years, and three molars over 2.5 years of age). Sampling was performed at the time of slaughter in the location where each animal was shot. Blood samples were collected from the thoracic cavity, into sterile 10 mL vacutainers containing clot activator, and sent to the Immunology Department, Scientific Institute of Veterinary Medicine of Serbia. After spontaneous coagulation and centrifugation (at 1500 g for 10 min), the sera were decanted and stored at −20 °C until further diagnostics. The sample size corresponded to the planned wild boar annual hunting bag that, with its range, exceeded a statistically representative sample as determined by statistical methods.

### 2.2. Serological Tests

Sera were analysed by two methods. A serum was considered positive when the two applied tests both showed positive reactions. The applied serology tests were RBT, used as a screening test, and c-ELISA, used as a confirmatory test. Antigen for RBT test was a suspension of inactivated *Brucella abortus* biovar 1 Weybridge strain No 99, obtained from ID.vet (Grabels, France). For this test, equal volumes (25 µL) of serum and antigen were mixed on a glass plate, forming a zone approximately 2 cm in diameter, gently stirred, and visualised for the presence of agglutination. The reaction was read after four minutes and any visible reaction was considered positive [7].

C-ELISA assay (SVANOVIR^®^, Brucella-Ab C-ELISA, Svanova, Uppsala, Sweden) was performed according to the manufacturer’s instructions. The optical density (OD) was measured using an ELISA reader (Tecan) at a single wave length of 450 nm. Sera were considered positive when the percent inhibition (PI value) was ≥30%. According to the manufacturer, diagnostic sensitivity and specificity values of the used ELISA kit were 99.5% and 99.6%, respectively.

### 2.3. Statistical Analysis

In the statistical analysis, we used descriptive statistical methods, Fisher’s exact test, risk ratio and prevalence estimation. We used Cohen’s Kappa test to assess the agreement between RBT and c-ELISA tests. Confidence intervals were calculated using the Wilson score method. Fisher’s exact test was used to determine the significance of the data. We also calculated the diagnostic parameters of the tests: negative and positive predictive value, expected number of true positive results, false negative and true negative results, the likelihood ratio for a positive result and likelihood ratio for a negative result, risk ratio and odds ratio (OR). In addition, we analysed the association of the age of wild boars with the seroprevalence of brucellosis. The strength of association between dependent and explanatory variables was estimated using ORs with 95% confidence intervals (95 CIs). Binary logistic regression (BLR) was used to model the relationship between predictors (age and sex) and dependent variables, i.e., seropositivity (for more information see Supplementary material, Table S1). The initial working hypothesis was that the age of wild boars was a risk factor for *Brucella* infection, so the hunted wild boars were stratified into four strata: up to 6 months of age, 6 to 18 months, 1.5 to 2.5 years, and boars older than 2.5 years. Within each stratum formed, we determined the seroprevalence. The statistical analyses were done using the statistical software packages IBM SPSS v. 26.0.0.0, XLSTAT Version 2014.5.03. and OpenEpi Version 3 (https://www.openepi.com/Menu/OE_Menu.htm (accessed on 17 June 2022).

## 3. Results

In total, 480 blood samples were collected from 304 (63.33%) male and 176 (36.66%) female individuals. The collected sera were examined for the presence of anti-*Brucella* antibodies, out of which 45 tested positive. The overall apparent seroprevalence of *Brucella* infection was 9.4% and the true prevalence was 9.06% bounded by 95 CIs of 6.74% and 12.02%. Seroprevalence data of *Brucella* in wild boars in Serbia are presented in Table 1. The greatest percentages of *Brucella* seropositive animals were detected mainly in the eastern parts of the country and in one of the central districts, i.e., Pomoravski, Branicevski, Borski and Juznobanatski.

Following the expected sensitivity and specificity of the tests as given by the manufacturers, RBT and c-ELISA should classify correctly 98.96% and 99.58% of samples, respectively. RBT should misclassify 1.04% of samples (two false-negative results and three false-positive results, while c-ELISA only 0.42% (two false-negative results and one false-positive result). Table 2 and Table 3 show the properties of the RBT and c-ELISA tests used in the study.

The two applied serology test methods showed some deviation, as 50 sera were positive according to RBT, while out of these, 45 sera with positive reactions were confirmed by the c-ELISA test. Sera were only considered as positive when both of the two applied tests gave positive reactions. As shown in Table 4 and Table 5, RBT and c-ELISA tests showed excellent, almost perfect mutual agreement (Cohen’s Kappa test 0.94).

The majority of tested animals were 6–18 months old (46.5%), while the categories 0–6 months, 1.5–2.5 years and older than 2.5 years contained 2.2%, 32.1% and 19.1% of the animals, respectively. *Brucella* seropositive wild boars belonged to the age categories 0–6 months (n = 3), 6–18 months (n = 14), 1.5–2.5 years (n = 23) and older than 2.5 years (n = 5). Table 6 shows the results of wild boar sera testing with the c-ELISA test, stratified by the age categories.

By comparing the seroprevalences in different age groups of wild boars, we found there were obvious differences between the age strata and that these differences were significant. The highest percentage of *Brucella* seropositive boars was found in the category of young animals, aged up to 6 months (27.12%), then in pigs aged 1.5 to 2.5 years (14.67%), then in pigs aged 6–18 months (5.93%), and the lowest in pigs older than 2.5 years (5.08%) (Table 7).

Table 8 and Table 9 show the values of the relative risks and risk differences in different age groups of wild boars tested for the presence of specific antibodies against *Brucella*.

*Brucella* seroprevalence differences between different age groups as a whole were statistically significant (Fisher’s exact test two-tailed *p* value = 0.0038). Wild boars aged 6–18 months have a 1.07-fold higher risk of having *Brucella* antibodies than wild boars over 2.5 years old, which makes a negligible difference between these two age categories. However, wild boars aged 1.5–2.5 years have a 2.75-fold higher risk of harbouring *Brucella* antibodies than wild boars over 2.5 years of age, whereas wild boars aged 1.5–2.5 years have a 2.56-fold higher risk of harbouring *Brucella* antibodies than wild boars aged 6–18 months. Risk ratio analysis indicated that wild boars between 1.5 and 2.5 years of age are at the greatest risk of infection.

**Figure 2:**
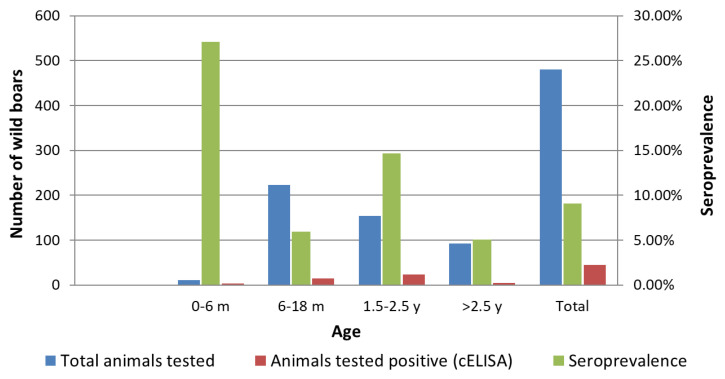
Distribution of the total tested wild boars, the number of *Brucella* seropositive individuals and the determined seroprevalence of brucellosis in different age groups of wild boars.

Figure 2 provides a comparative overview of the distribution of tested sera, the number of *Brucella* seropositive individuals and the established seroprevalence of brucellosis in different age groups of wild boars.

BLR analysis proved a negative and positive association between the different age groups of wild boars and seropositivity, i.e., the registered number of *Brucella* seropositive wild boars compared to the youngest analysed category (age 6–18 months) as a reference category. The regression coefficient, B, for the model predictor variables was positive and significant for age group 1.5–2.5 years, indicating that this age group is more susceptible to infection than age group 6 to 18 months, which was not the case with the age group over 2.5 years where the regression coefficient, B, was negative and not significant. The regression coefficients for the different age groups of wild boars are given in Table 10. Regarding the OR, an exponential value of B of 2.613 for the age group 1.5–2.5 years indicates this age group is 2.613 times more likely to be infected with *Brucella* than the age group 6–18 months (Table 10; Supplementary material, Tables S1–S4; Supplementary material, Figures S1 and S2).

Table 11 shows the results of mutual comparisons of the significance of the differences of different age groups and different sexes of wild boars. The sex of the boars was not a significant predictor of seropositivity (regression coefficient *p* = 0.358) (Table 11).

## 4. Discussion

Brucellosis is a significant bacterial disease causing direct production losses in domestic animals, and thus, it is constantly highly ranked among the most economically important zoonoses worldwide. During the 1990s, due to armed conflict and uncontrolled movement of infected sheep, brucellosis spread to different parts of Serbia [19]. Recently, brucellosis in Serbia in domestic pigs has been sporadically confirmed, mostly in backyard farmed pigs after reproductive disorders were reported [20], while a serosurvey covering the period 2011–2015 detected 88 *Brucella* seropositive domestic pigs [21]. To date, no studies regarding *Brucella* infection in wild boars in Serbia have been published.

The prevalence of brucellosis has noticeably increased in many countries where pigs exist together with an overabundance of wildlife. In parallel with the growth of the wild boar population, in most countries, the prevalence of brucellosis has increased [15]. The population of wild boars in Serbia in recent years has remained reasonably constant, and in 2019, the population size was about 25,536 individuals [22]. To date, the presence in Serbia of *B. melitensis*, *B. suis* biovar 2 and *B. canis* has been reported [17]. *B. suis* biovar 2 was confirmed in two aborted foetuses from outdoor reared pigs in the northwestern part of the country, Srem, a mostly forested plains district, was reported by Zutic et al. [23]. The authors assumed that direct or indirect contact with wild boars led to the infection. Rogozarski et al. [24] isolated the same biovar from the epididymal puncture of a boar affected with brucellosis.

Our study revealed the true seroprevalence of *Brucella* infection in the wild boar population of 9.06%, and shows the first published data regarding *Brucella* seropositivity in the wild boar population in Serbia, to date. The presence of *Brucella* in the wild boar population in Serbia was not unexpected since this microorganism has been confirmed in neighbouring countries, where *B. suis* biovar 2 was predominantly isolated [25,26]. Moreover, Cvetnic et al. [14] confirmed the presence of *B. suis* biovar 3 in wild boars.

A global comprehensive literature review and meta-analysis of *Brucella* in pigs from 2000 to 2020 reported that the overall prevalence of brucellosis in wild boars between 2006 and 2010 was 22.3% [27], while after 2010, the prevalence gradually decreased after the World Organisation for Animal Health (WOAH) proposed control safety standards for animal production [28]. *De facto*, the *Brucella* seroprevalence in wild boars in Croatia varied through the years, ranging from 1.3% to 27.6% during different periods [13,14,25]. These data are in accordance with our obtained results, showing a *Brucella* seroprevalence of 9.06%. In our study, by comparing the *Brucella* seroprevalence in different age groups of wild boar, we found that these differences are statistically significant. Based on the BLR analysis, it was established that the age group of 1.5–2.5 years is the most susceptibile to infection, which was not the case with the age group over 2.5 years. Those findings are in line with the fact the greatest number and percentage of *Brucella* seropositive animals detected in the current study were in the age category 1.5–2.5 years. The findings are also consistent with the fact that the majority of wild boars reach puberty and start to mate within the first year of life, which favours the spread of infection. On the other hand, of 11 tested piglets aged 0–6 months, three were *Brucella* seropositive. This high seroprevalence in pigs aged 0–6 months cannot be reliably explained. However, taking into account biological plausibility and common modes of transmission and routes of infection, influencing factors could be maternal antibodies, piglets being vertically infected [29] and false-positive serological results due to *Yersinia enterocolitica* O:9 infection causing cross-reactivity. Analysis showed the sex of the boars is not a significant predictor of seropositivity.

A limitation of this study was the significantly smaller number of sera tested in the young age stratum compared to other age strata (total of 11 sera in the <6 month stratum). Due to this limitation in terms of sample size tested in this category of very young animals, and also due to the possible influence of unknown confounders, we concluded that it would be unjustifiable to include this stratum in the further statistical and epizootiological analyses. In that sense, no statistical comparisons were made of this stratum with other strata, i.e., other older categories of wild boars.

Both serological tests used in the study proved to be reliable for this type of study, especially when used in tandem.

Brucellosis can be a permanent risk even in the face of continuously applied monitoring programs, not only for domestic pigs, but for other domestic animals and humans. Since Serbia is not recognized as being officially free of brucellosis, legislation on brucellosis in Serbia prescribes mandatory serology testing for cattle older than 12 months, for sheep and goats older than 6 months, and for all breeding boars, rams and bulls kept either for natural breeding or artificial insemination [30]. Furthermore, every abortion in sows must be notified and examined for brucellosis [30]. Long-term brucellosis surveillance is well-regulated for domestic animals, but in contrast, brucellosis monitoring programs in wildlife are not conducted.

Our study indicates that wild boars are a very likely reservoir of *Brucella* for domestic pigs, considering pigs are frequently reared outdoors with low biosafety measures applied. These circumstances allow close contact of domestic and wild pigs, often resulting in them mating. In addition, the risk factors for transmission of the pathogen between wild boars and outdoor-reared pigs are linked to the presence and density of wild boar and domestic pig populations, disease prevalence, the features of the inhabited regions, fence characteristics etc. [31]. Wild boar meat has recently become more popular, and harvesting this product facilitates pathogen transmission [32]. Moreover, we hypothesise that the wild boar population is growing, and this has likely led to more frequent free movement of wild boars near commercial pig farms, perhaps increasing the risk of infection transmission to domestic pigs.

The greatest numbers of *Brucella* seropositive animals were detected in the eastern parts of the country and in one of the central districts, i.e., Pomoravski, Branicevski, Borski and Juznobanatski. The most likely reason for higher seropositivity in certain areas is the higher density of wild boars living in these localities. Additionally intriguing is the fact that *Brucella* seropositive wild boars were found in eastern parts of the country, i.e., border areas with neighbouring countries Hungary and Bulgaria, indicating the need for further, extended studies that will cover other administrative districts and include more animals. Therefore, cooperation between countries in the region is essential and should provide a better understanding of the epizootiological situation, since genetic similarity among circulating *Brucella* strains in the population of domestic and wild pigs originating from Hungary, Croatia and Germany was observed, highlighting the inability of state borders to control the spread of the pathogen [26,33].

The complexity of interactions among animals and humans is evident, and so the global picture of brucellosis remains incomplete, while in wild boars the disease is insufficiently clarified. Since the disease has recently re-emerged as a public health concern, brucellosis should be considered thoughtfully and disease control measures should be constantly maintained. Additional, comprehensive studies regarding *Brucella* biological characteristics, epizootiology and risk of transmission to domestic pigs and humans are in crucial need.

## Figures and Tables

**Table 1:** Seroprevalence of *Brucella* in wild boars in Serbia.

Administrative District	Year	Analysed Sera	Positive Sera	%
Branicevski	2020	43	5	11.6
	2021	139	18	12.9
Borski	2020	22	2	9.1
	2021	17	1	5.9
Zajecarski	2020	23	0	0
	2021	41	0	0
Pomoravski	2020	32	7	21.9
	2021	12	3	25.0
Juznobanatski	2020	38	1	2.6
	2021	101	8	7.9
Sumadijski	2020	0	0	0
	2021	12	0	0
Total		480	45	9.4

**Table 2:** Properties of RBT.

Statistic	Value	Lower Bound (95%)	Upper Bound (95%)
Correct classification	98.96%	98.05%	99.87%
Misclassification	1.04%	0.13%	1.95%
Sensitivity	96.10% *	86.52%	98.95%
Specificity	99.30% *	97.97%	99.76%
False positive rate	0.70%	0.24%	2.03%
False negative rate	3.91%	2.45%	6.18%
PPV (Positive Predictive Value)	93.96%	83.73%	97.92%
NPV (Negative Predictive Value)	99.56%	98.35%	99.88%
LR+ (Positive likelihood ratio)	137.15	44.52	137.17
LR- (Negative likelihood ratio)	0.04	0.01	0.16
Diagnostic odds ratio	3486.65	555.49	21,884.54

* Data available in Reference [18].

**Table 3:** Properties of c-ELISA test.

Statistic	Value	Lower Bound (95%)	Upper Bound (95%)
Correct classification	99.58%	99.01%	100.00%
Misclassification	0.42%	0.00%	0.99%
Sensitivity	99.50%	90.98%	99.97%
Specificity	99.60%	98.43%	99.90%
False positive rate	0.40%	0.10%	1.57%
False negative rate	0.50%	0.15%	1.73%
PPV (Positive Predictive Value)	96.11%	85.96%	99.01%
NPV (Negative Predictive Value)	99.95%	99.03%	100.00%
LR+ (Positive likelihood ratio)	248.18	56.57	248.18
LR- (Negative likelihood ratio)	0.0051	0.0001	0.3282
Diagnostic odds ratio	48,842.17	573.53	4,159,440.17

**Table 4:** c-ELISA vs. RBT cross tabulation.

Laboratory Test			RBT		Total
			T-	T+	
c-ELISA	T-	Count	430	5	435
		Expected Count	389.7	45.3	435.0
		% within c-ELISA	98.9%	1.1%	100.0%
		% within RBT	100.0%	10.0%	90.6%
	T+	Count	0	45	45
		Expected Count	40.3	4.7	45.0
		% within c-ELISA	0.0%	100.0%	100.0%
		% within RBT	0.0%	90.0%	9.4%
Total		Count	430	50	480
		Expected Count	430	50	480
		% within c-ELISA	89.6%	10.4%	100.0%
		% within RBT	100.0%	100.0%	100.0%

**Table 5:** Test agreement of RBT and c-ELISA (Cohen’s Kappa test).

	Statistic	Value	Asymptotic Standard Error	Approximated Significance
Measure of Agreement	Kappa	0.94	0.026	0.000
Num. of Valid Cases		480		

**Table 6:** The results of c-ELISA stratified by age.

Test Result	Age of Wild Boar			
	0–6 m	6–18 m	1.5–2.5 y	>2.5 y
T+	3	14	23	5
T-	8	209	131	87
Total	11	223	154	92

**Table 7:** Seroprevalence of brucellosis in wild boars stratified by age.

Age	Total Animals Tested	Positive Samples	Seroprevalence	Confidence Limits	
				LL	UL
0–6 m	11	3	27.12%	9.43%	56.67%
6–18 m	223	14	5.93%	3.41%	9.95%
1.5–2.5 y	154	23	14.67%	9.85%	21.20%
>2.5 y	92	5	5.08%	1.96%	11.80%
Total	480	45	9.06%	6.74%	12.02%

**Table 8:** The relative risk of brucellosis in different age groups of wild boars.

Age Groups	Relative Risk	Confidence Limits		*p*-Value
		LL	UL	
1.5–2.5 y vs. 6–18 m	2.56	1.34	4.90	0.0045
6–18 m vs. >2.5 y	1.07	0.39	2.92	0.8909
1.5–2.5 y vs. >2.5 y	2.75	1.08	6.98	0.0335

**Table 9:** Risk differences of brucellosis between different age groups of wild boars.

Age Groups	Risk Difference	Confidence Limits		*p*-Value
		LL	UL	
1.5–2.5 y vs. 6–18 m	9.11%	2.69%	15.52%	0.0031
6–18 m vs. >2.5 y	0.39%	−5.17%	5.96%	0.0891
1.5–2.5 y vs. >2.5 y	9.50%	2.21%	16.79%	0.0235

**Table 10:** Results of the regression analysis (predicted variable: wild boar age; explanatory variable: *Brucella* seroprevalence).

Source	B	SEE	Wald	Sig. (*p*)	Odds Ratio (OR)	95% C.I. for Exp(B)	
						Lower	Upper
Intercept	−2.708	0.276	96.253	<0.0001			
Age 6–18 m	0.000	0.000					
Age 1.5–2.5 y	0.961	0.357	7.254	0.0071	2.6136	1.2990	5.2587
Age >2.5 y	−0.160	0.536	0.089	0.7656	0.8523	0.2979	2.4380

**Table 11:** Results of mutual comparisons of the significance of the differences of different age groups of wild boars and different sexes.

Contrast	DF	Chi-Square	*p* > Chi^2^
Age-6–18 m vs. Age-1.5–2.5 y	1	7.4001	0.007
Age-6–18 m vs. Age->2.5 y	1	0.0861	0.769
Age-1.5–2.5 y vs. Age->2.5 y	1	4.8517	0.028
Sex-male vs. Sex-female	1	0.8462	0.358

## Data Availability

Not applicable.

## Supplementary Materials

The following supporting information can be downloaded at: https://www.mdpi.com/article/10.3390/vetsci9100575/s1, Table S1: Age structure, gender of investigated wild boar population and results of ELISA test (detection of *Brucella* specific antibodies); Table S2: Summery statistics; Table S3: Goodness of fit statistics (Variable ELISA); Table S4: Standardized coefficients (Variable ELISA); Figure S1: Standardized coefficients (Variable ELISA); Figure S2: ROC Curve (Variable ELISA) AUC- Area under the curve.
