# Supplementary material for: First Report of Brucella Seroprevalence in Wild Boar Population in Serbia
Source: Vet Sci. 2022 Oct 17;9(10):575. doi: 10.3390/vetsci9100575 (PMC9612294; doi:10.3390/vetsci9100575)
Supplement: Supplementary file 1 [file vetsci-09-00575-s001.zip › vetsci-1899084-supplementary.pdf]

Supplementary materials

Table S1. Age structure, gender of investigated wild boar population and results of ELISA test (detection of *Brucella* specific antibodies).

Age	gender	ELISA	Age	gender	ELISA	6-18m	M	0
0-6m	M	1	6-18m	M	0	6-18m	M	0
0-6m	F	1	6-18m	M	0	6-18m	M	0
0-6m	F	1	6-18m	M	0	6-18m	M	0
0-6m	M	0	6-18m	M	0	6-18m	M	0
0-6m	M	0	6-18m	M	0	6-18m	M	0
0-6m	M	0	6-18m	M	0	6-18m	M	0
0-6m	M	0	6-18m	M	0	6-18m	M	0
0-6m	M	0	6-18m	M	0	6-18m	M	0
0-6m	F	0	6-18m	M	0	6-18m	M	0
0-6m	F	0	6-18m	M	0	6-18m	M	0
0-6m	F	0	6-18m	M	0	6-18m	M	0
0-6m	F	0	6-18m	M	0	6-18m	M	0
6-18m	M	1	6-18m	M	0	6-18m	M	0
6-18m	M	1	6-18m	M	0	6-18m	M	0
6-18m	M	1	6-18m	M	0	6-18m	M	0
6-18m	M	1	6-18m	M	0	6-18m	M	0
6-18m	M	1	6-18m	M	0	6-18m	M	0
6-18m	M	1	6-18m	M	0	6-18m	M	0
6-18m	M	1	6-18m	M	0	6-18m	M	0
6-18m	M	1	6-18m	M	0	6-18m	M	0
6-18m	M	1	6-18m	M	0	6-18m	M	0
6-18m	F	1	6-18m	M	0	6-18m	M	0
6-18m	F	1	6-18m	M	0	6-18m	M	0
6-18m	F	1	6-18m	M	0	6-18m	M	0
6-18m	F	1	6-18m	M	0	6-18m	M	0
6-18m	F	1	6-18m	M	0	6-18m	M	0
6-18m	M	0	6-18m	M	0	6-18m	M	0
6-18m	M	0	6-18m	M	0	6-18m	M	0
6-18m	M	0	6-18m	M	0	6-18m	M	0
6-18m	M	0	6-18m	M	0	6-18m	M	0
6-18m	M	0	6-18m	M	0	6-18m	M	0
6-18m	M	0	6-18m	M	0	6-18m	M	0
6-18m	M	0	6-18m	M	0	6-18m	M	0
6-18m	M	0	6-18m	M	0	6-18m	M	0
6-18m	M	0	6-18m	M	0	6-18m	M	0
6-18m	M	0	6-18m	M	0	6-18m	M	0
6-18m	M	0	6-18m	M	0	6-18m	M	0

Continuation of Table S1.

Age	gender	ELISA	Age	gender	ELISA	6-18m	M	0
6-18m	M	0	6-18m	F	0	6-18m	F	0
6-18m	M	0	6-18m	F	0	6-18m	F	0
6-18m	M	0	6-18m	F	0	6-18m	F	0
6-18m	M	0	6-18m	F	0	6-18m	F	0
6-18m	M	0	6-18m	F	0	6-18m	F	0
6-18m	M	0	6-18m	F	0	6-18m	F	0
6-18m	M	0	6-18m	F	0	6-18m	F	0
6-18m	M	0	6-18m	F	0	6-18m	F	0
6-18m	M	0	6-18m	F	0	6-18m	F	0
6-18m	M	0	6-18m	F	0	6-18m	F	0
6-18m	M	0	6-18m	F	0	6-18m	F	0
6-18m	M	0	6-18m	F	0	6-18m	F	0
6-18m	M	0	6-18m	F	0	6-18m	F	0
6-18m	M	0	6-18m	F	0	6-18m	F	0
6-18m	M	0	6-18m	F	0	6-18m	F	0
6-18m	M	0	6-18m	F	0	6-18m	F	0
6-18m	M	0	6-18m	F	0	6-18m	F	0
6-18m	M	0	6-18m	F	0	6-18m	F	0
6-18m	M	0	6-18m	F	0	6-18m	F	0
6-18m	M	0	6-18m	F	0	6-18m	F	0
6-18m	M	0	6-18m	F	0	6-18m	F	0
6-18m	M	0	6-18m	F	0	6-18m	F	0
6-18m	M	0	6-18m	F	0	6-18m	F	0
6-18m	M	0	6-18m	F	0	6-18m	F	0
6-18m	M	0	6-18m	F	0	6-18m	F	0
6-18m	M	0	6-18m	F	0	6-18m	F	0
6-18m	M	0	6-18m	F	0	6-18m	F	0
6-18m	M	0	6-18m	F	0	6-18m	F	0
6-18m	M	0	6-18m	F	0	6-18m	F	0
6-18m	M	0	6-18m	F	0	6-18m	F	0
6-18m	M	0	6-18m	F	0	6-18m	F	0
6-18m	M	0	6-18m	F	0	1.5-2.5y	M	1
6-18m	M	0	6-18m	F	0	1.5-2.5y	M	1
6-18m	M	0	6-18m	F	0	1.5-2.5y	M	1
6-18m	M	0	6-18m	F	0	1.5-2.5y	M	1
6-18m	M	0	6-18m	F	0	1.5-2.5y	M	1
6-18m	M	0	6-18m	F	0	1.5-2.5y	M	1

Continuation of Table S1.

Age	gender	ELISA	Age	gender	ELISA	6-18m	M	0
1.5-2.5y	M	1	1.5-2.5y	M	0	1.5-2.5y	M	0
1.5-2.5y	M	1	1.5-2.5y	M	0	1.5-2.5y	M	0
1.5-2.5y	M	1	1.5-2.5y	M	0	1.5-2.5y	M	0
1.5-2.5y	M	1	1.5-2.5y	M	0	1.5-2.5y	M	0
1.5-2.5y	M	1	1.5-2.5y	M	0	1.5-2.5y	M	0
1.5-2.5y	M	1	1.5-2.5y	M	0	1.5-2.5y	M	0
1.5-2.5y	M	1	1.5-2.5y	M	0	1.5-2.5y	M	0
1.5-2.5y	M	1	1.5-2.5y	M	0	1.5-2.5y	M	0
1.5-2.5y	M	1	1.5-2.5y	M	0	1.5-2.5y	M	0
1.5-2.5y	M	1	1.5-2.5y	M	0	1.5-2.5y	M	0
1.5-2.5y	M	1	1.5-2.5y	M	0	1.5-2.5y	M	0
1.5-2.5y	F	1	1.5-2.5y	M	0	1.5-2.5y	M	0
1.5-2.5y	F	1	1.5-2.5y	M	0	1.5-2.5y	M	0
1.5-2.5y	F	1	1.5-2.5y	M	0	1.5-2.5y	M	0
1.5-2.5y	F	1	1.5-2.5y	M	0	1.5-2.5y	M	0
1.5-2.5y	F	1	1.5-2.5y	M	0	1.5-2.5y	M	0
1.5-2.5y	F	1	1.5-2.5y	M	0	1.5-2.5y	M	0
1.5-2.5y	M	0	1.5-2.5y	M	0	1.5-2.5y	M	0
1.5-2.5y	M	0	1.5-2.5y	M	0	1.5-2.5y	M	0
1.5-2.5y	M	0	1.5-2.5y	M	0	1.5-2.5y	M	0
1.5-2.5y	M	0	1.5-2.5y	M	0	1.5-2.5y	M	0
1.5-2.5y	M	0	1.5-2.5y	M	0	1.5-2.5y	M	0
1.5-2.5y	M	0	1.5-2.5y	M	0	1.5-2.5y	M	0
1.5-2.5y	M	0	1.5-2.5y	M	0	1.5-2.5y	M	0
1.5-2.5y	M	0	1.5-2.5y	M	0	1.5-2.5y	M	0
1.5-2.5y	M	0	1.5-2.5y	M	0	1.5-2.5y	M	0
1.5-2.5y	M	0	1.5-2.5y	M	0	1.5-2.5y	M	0
1.5-2.5y	M	0	1.5-2.5y	M	0	1.5-2.5y	M	0
1.5-2.5y	M	0	1.5-2.5y	M	0	1.5-2.5y	M	0
1.5-2.5y	M	0	1.5-2.5y	M	0	1.5-2.5y	M	0
1.5-2.5y	M	0	1.5-2.5y	M	0	1.5-2.5y	M	0
1.5-2.5y	M	0	1.5-2.5y	M	0	1.5-2.5y	M	0
1.5-2.5y	M	0	1.5-2.5y	M	0	1.5-2.5y	M	0
1.5-2.5y	M	0	1.5-2.5y	M	0	1.5-2.5y	M	0
1.5-2.5y	M	0	1.5-2.5y	M	0	1.5-2.5y	M	0
1.5-2.5y	M	0	1.5-2.5y	M	0	1.5-2.5y	M	0
1.5-2.5y	M	0	1.5-2.5y	M	0	1.5-2.5y	M	0

Continuation of Table S1.

Age	gender	ELISA	Age	gender	ELISA	6-18m	M	0
1.5-2.5y	M	0	1.5-2.5y	M	0	1.5-2.5y	M	0
1.5-2.5y	M	0	1.5-2.5y	M	0	1.5-2.5y	M	0
1.5-2.5y	M	0	1.5-2.5y	M	0	1.5-2.5y	M	0
1.5-2.5y	M	0	1.5-2.5y	M	0	1.5-2.5y	M	0
1.5-2.5y	M	0	1.5-2.5y	M	0	1.5-2.5y	M	0
1.5-2.5y	M	0	1.5-2.5y	M	0	1.5-2.5y	M	0
1.5-2.5y	M	0	1.5-2.5y	M	0	1.5-2.5y	M	0
1.5-2.5y	M	0	1.5-2.5y	M	0	1.5-2.5y	M	0
1.5-2.5y	M	0	1.5-2.5y	M	0	1.5-2.5y	M	0
1.5-2.5y	M	0	1.5-2.5y	M	0	1.5-2.5y	M	0
1.5-2.5y	M	0	1.5-2.5y	M	0	1.5-2.5y	M	0
1.5-2.5y	F	0	1.5-2.5y	F	0	1.5-2.5y	F	0
1.5-2.5y	F	0	1.5-2.5y	F	0	1.5-2.5y	F	0
1.5-2.5y	F	0	1.5-2.5y	F	0	1.5-2.5y	F	0
1.5-2.5y	F	0	1.5-2.5y	F	0	1.5-2.5y	F	0
1.5-2.5y	F	0	1.5-2.5y	F	0	1.5-2.5y	F	0
1.5-2.5y	F	0	1.5-2.5y	F	0	1.5-2.5y	F	0
1.5-2.5y	F	0	1.5-2.5y	F	0	1.5-2.5y	F	0
1.5-2.5y	F	0	1.5-2.5y	F	0	1.5-2.5y	F	0
1.5-2.5y	F	0	1.5-2.5y	F	0	1.5-2.5y	F	0
1.5-2.5y	F	0	1.5-2.5y	F	0	1.5-2.5y	F	0
1.5-2.5y	F	0	1.5-2.5y	F	0	1.5-2.5y	F	0
1.5-2.5y	F	0	1.5-2.5y	F	0	1.5-2.5y	F	0
1.5-2.5y	F	0	1.5-2.5y	F	0	1.5-2.5y	F	0
1.5-2.5y	F	0	1.5-2.5y	F	0	1.5-2.5y	F	0
1.5-2.5y	F	0	1.5-2.5y	F	0	1.5-2.5y	F	0
1.5-2.5y	F	0	1.5-2.5y	F	0	1.5-2.5y	F	0
1.5-2.5y	F	0	1.5-2.5y	F	0	1.5-2.5y	F	0
1.5-2.5y	F	0	1.5-2.5y	F	0	1.5-2.5y	F	0
1.5-2.5y	F	0	1.5-2.5y	F	0	1.5-2.5y	F	0
1.5-2.5y	F	0	1.5-2.5y	F	0	1.5-2.5y	F	0
1.5-2.5y	F	0	1.5-2.5y	F	0	1.5-2.5y	F	0
1.5-2.5y	F	0	1.5-2.5y	F	0	1.5-2.5y	F	0
1.5-2.5y	F	0	1.5-2.5y	F	0	1.5-2.5y	F	0
1.5-2.5y	F	0	1.5-2.5y	F	0	1.5-2.5y	F	0
1.5-2.5y	F	0	1.5-2.5y	F	0	1.5-2.5y	F	0
1.5-2.5y	F	0	1.5-2.5y	F	0	1.5-2.5y	F	0
1.5-2.5y	F	0	1.5-2.5y	F	0	1.5-2.5y	F	0

Continuation of Table S1.

Age	gender	ELISA	Age	gender	ELISA	6-18m	M	0
1.5-2.5y	F	0	1.5-2.5y	F	0	>2.5y	M	0
1.5-2.5y	F	0	1.5-2.5y	F	0	>2.5y	M	0
1.5-2.5y	F	0	1.5-2.5y	F	0	>2.5y	M	0
1.5-2.5y	F	0	1.5-2.5y	F	0	>2.5y	M	0
1.5-2.5y	F	0	1.5-2.5y	F	0	>2.5y	M	0
1.5-2.5y	F	0	1.5-2.5y	F	0	>2.5y	M	0
1.5-2.5y	F	0	1.5-2.5y	F	0	>2.5y	M	0
1.5-2.5y	F	0	1.5-2.5y	F	0	>2.5y	M	0
1.5-2.5y	F	0	1.5-2.5y	F	0	>2.5y	M	0
1.5-2.5y	F	0	1.5-2.5y	F	0	>2.5y	M	0
1.5-2.5y	F	0	1.5-2.5y	F	0	>2.5y	M	0
1.5-2.5y	F	0	1.5-2.5y	F	0	>2.5y	M	0
1.5-2.5y	F	0	1.5-2.5y	F	0	>2.5y	M	0
1.5-2.5y	F	0	1.5-2.5y	F	0	>2.5y	M	0
1.5-2.5y	F	0	1.5-2.5y	F	0	>2.5y	M	0
1.5-2.5y	F	0	1.5-2.5y	F	0	>2.5y	M	0
1.5-2.5y	F	0	1.5-2.5y	F	0	>2.5y	M	0
1.5-2.5y	F	0	1.5-2.5y	F	0	>2.5y	M	0
1.5-2.5y	F	0	1.5-2.5y	F	0	>2.5y	M	0
1.5-2.5y	F	0	1.5-2.5y	F	0	>2.5y	M	0
1.5-2.5y	F	0	1.5-2.5y	F	0	>2.5y	M	0
1.5-2.5y	F	0	1.5-2.5y	F	0	>2.5y	M	0
1.5-2.5y	F	0	1.5-2.5y	F	0	>2.5y	M	0
>2.5y	M	1	>2.5y	M	1	>2.5y	M	0
>2.5y	M	1	>2.5y	M	1	>2.5y	M	0
>2.5y	M	1	>2.5y	M	1	>2.5y	M	0
>2.5y	F	1	>2.5y	F	1	>2.5y	M	0
>2.5y	F	1	>2.5y	F	1	>2.5y	M	0
>2.5y	M	0	>2.5y	M	0	>2.5y	M	0
>2.5y	M	0	>2.5y	M	0	>2.5y	M	0
>2.5y	M	0	>2.5y	M	0	>2.5y	M	0
>2.5y	M	0	>2.5y	M	0	>2.5y	M	0
>2.5y	M	0	>2.5y	M	0	>2.5y	M	0
>2.5y	M	0	>2.5y	M	0	>2.5y	M	0
>2.5y	M	0	>2.5y	M	0	>2.5y	M	0
>2.5y	M	0	>2.5y	M	0	>2.5y	M	0
>2.5y	M	0	>2.5y	M	0	>2.5y	M	0

Continuation of Table S1.

Age	gender	ELISA
>2.5y	F	0
>2.5y	F	0
>2.5y	F	0
>2.5y	F	0
>2.5y	F	0
>2.5y	F	0
>2.5y	F	0
>2.5y	F	0
>2.5y	F	0
>2.5y	F	0
>2.5y	F	0
>2.5y	F	0
>2.5y	F	0
>2.5y	F	0
>2.5y	F	0
>2.5y	F	0
>2.5y	F	0
>2.5y	F	0
>2.5y	F	0
>2.5y	F	0
>2.5y	F	0
>2.5y	F	0
>2.5y	F	0
>2.5y	F	0
>2.5y	F	0
>2.5y	F	0
>2.5y	F	0
>2.5y	F	0
>2.5y	F	0
>2.5y	F	0
>2.5y	F	0
>2.5y	F	0
>2.5y	F	0
>2.5y	F	0
>2.5y	F	0
>2.5y	F	0
>2.5y	F	0

Results of regression analysis:

Table S2. Summery statistics

Variable	Categories	Frequencies	%
ELISA	0	430	91.1017
	1	42	8.8983

Variable	Categories	Frequencies	%
Age	6-18m	224	47.4576
	1.5-2.5y	155	32.8390
	>2.5y	93	19.7034
gender	M	298	63.1356
	F	174	36.8644

Table S3. Goodness of fit statistics (Variable ELISA)

Statistic	Independent	Full
Observations	472	472
Sum of weights	472.0000	472.0000
DF	471	468
-2 Log(Likelihood)	283.3686	272.9932
R2(McFadden)	0.0000	0.0366
R2(Cox and Snell)	0.0000	0.0217
R2(Nagelkerke)	0.0000	0.0482
AIC	285.3686	280.9932
SBC	289.5256	297.6211
Iterations	0	6

Table S4. Standardized coefficients (Variable ELISA)

Source	Value	Standard error	Wald Chi-Square	Pr > Chi2	Wald Lower bound (95%)	Wald Upper bound (95%)
Age-6-18m	0.0000	0.0000				
Age-1.5-2.5y	0.2488	0.0924	7.2540	0.0071	0.0677	0.4298
Age->2.5y	-0.0351	0.1176	0.0889	0.7656	-0.2655	0.1954

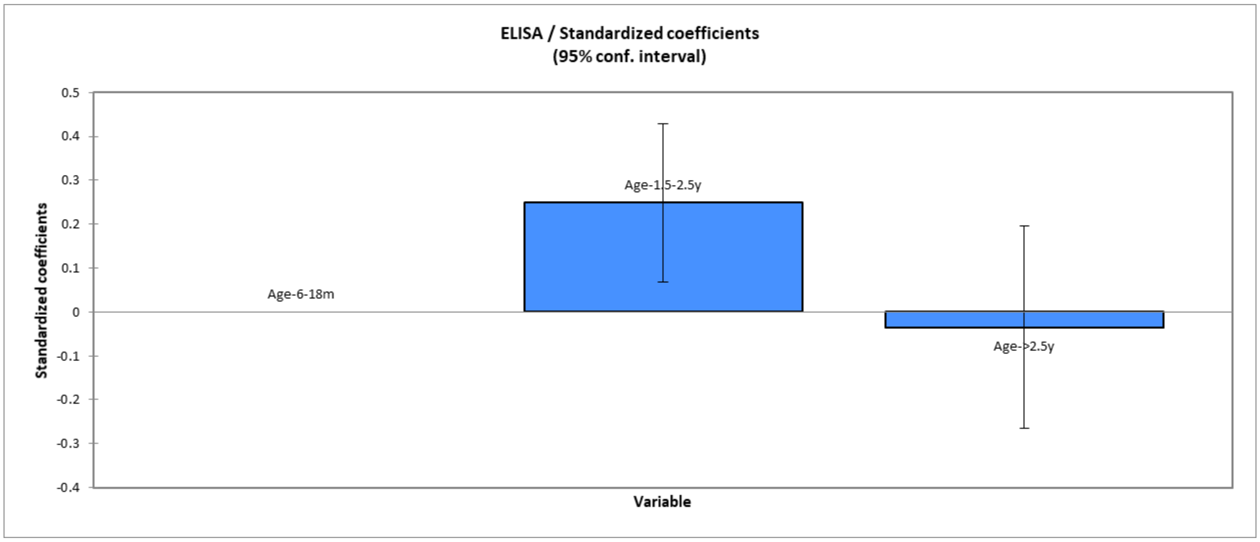

Figure S1. Standardized coefficients (Variable ELISA)

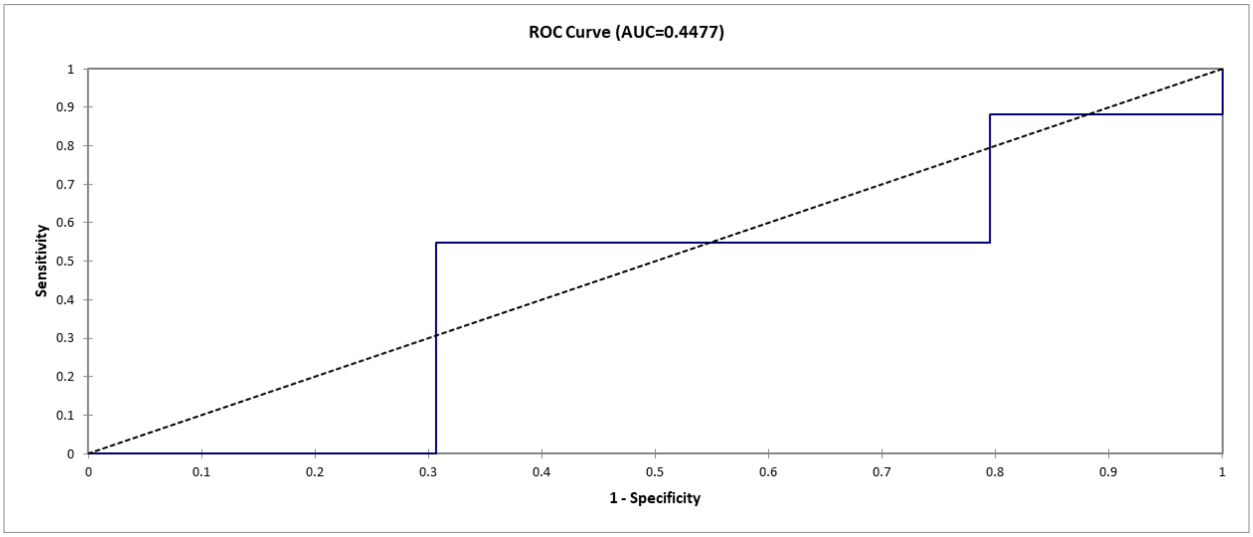

Figure S2. ROC Curve (Variable ELISA). AUC- Area under the curve
